# Supplementary material for: Effect of Vitamin B12 Replacement Intervals on Clinical Symptoms and Laboratory Findings in Gastric Cancer Patients after Total Gastrectomy
Source: Cancers (Basel). 2023 Oct 11;15(20):4938. doi: 10.3390/cancers15204938 (PMC10605534; doi:10.3390/cancers15204938)
Supplement: Supplementary file 1 [file cancers-15-04938-s001.zip › Supplementary Table 1_final.docx]

**Supplementary Table 1.** Vitamin B12 deficiency symptoms before and after vitamin B12 replacement.

| **Variable** | **Routine replacement group (**n **= 110)** | **Interval replacement group**  **(**n **= 80)** | **Lab-based replacement group**  **(**n **= 216)** | ***p*-value** |
| --- | --- | --- | --- | --- |
| Symptoms at pre-replacement | n = 110 | n = 80 | n = 216 | <0.001 |
| Absence | 65 (59.1) | 68 (85.0) | 174 (80.6) |  |
| Presence | 45 (40.9) | 12 (15.0) | 42 (19.4) |  |
| Symptoms 6 months after replacement | n = 105 | n = 77 | n = 211 | 0.025 |
| Absence | 83 (79.0) | 72 (93.5) | 188 (89.1) |  |
| Presence | 22 (21.0) | 5 (6.5) | 23 (10.9) |  |
| Symptoms 12 months after replacement | n = 98 | n = 75 | n = 204 | 0.115 |
| Absence | 83 (84.7) | 70 (93.3) | 186 (91.2) |  |
| Presence | 15 (15.3) | 5 (6.7) | 18 (8.8) |  |
| Symptoms 18 months after replacement | n = 86 | n = 69 | n = 175 | 0.206 |
| Absence | 70 (81.4) | 63 (91.3) | 155 (88.6) |  |
| Presence | 16 (18.6) | 6 (8.7) | 20 (11.4) |  |
| Symptoms 24 months after replacement | n = 77 | n = 61 | n = 158 | 0.616 |
| Absence | 67 (87.0) | 57 (93.4) | 139 (88.0) |  |
| Presence | 10 (13.0) | 4 (6.6) | 19 (12.0) |  |
| Symptoms 30 months after replacement | n = 61 | n = 46 | n = 108 | 0.521 |
| Absence | 51 (83.6) | 41 (89.1) | 97 (89.8) |  |
| Presence | 10 (16.4) | 5 (10.9) | 11 (10.2) |  |
| Symptoms 36months after replacement | n = 53 | n = 42 | n = 100 | 0.73 |
| Absence | 46 (86.8) | 39 (92.9) | 91 (91.0) |  |
| Presence | 7 (13.2) | 3 (7.1) | 9 (9.0) |  |

Values are n (%)
