# Supplementary figures and images for: Effect of Vitamin B12 Replacement Intervals on Clinical Symptoms and Laboratory Findings in Gastric Cancer Patients after Total Gastrectomy
Source: Cancers (Basel). 2023 Oct 11;15(20):4938. doi: 10.3390/cancers15204938 (PMC10605534; doi:10.3390/cancers15204938)

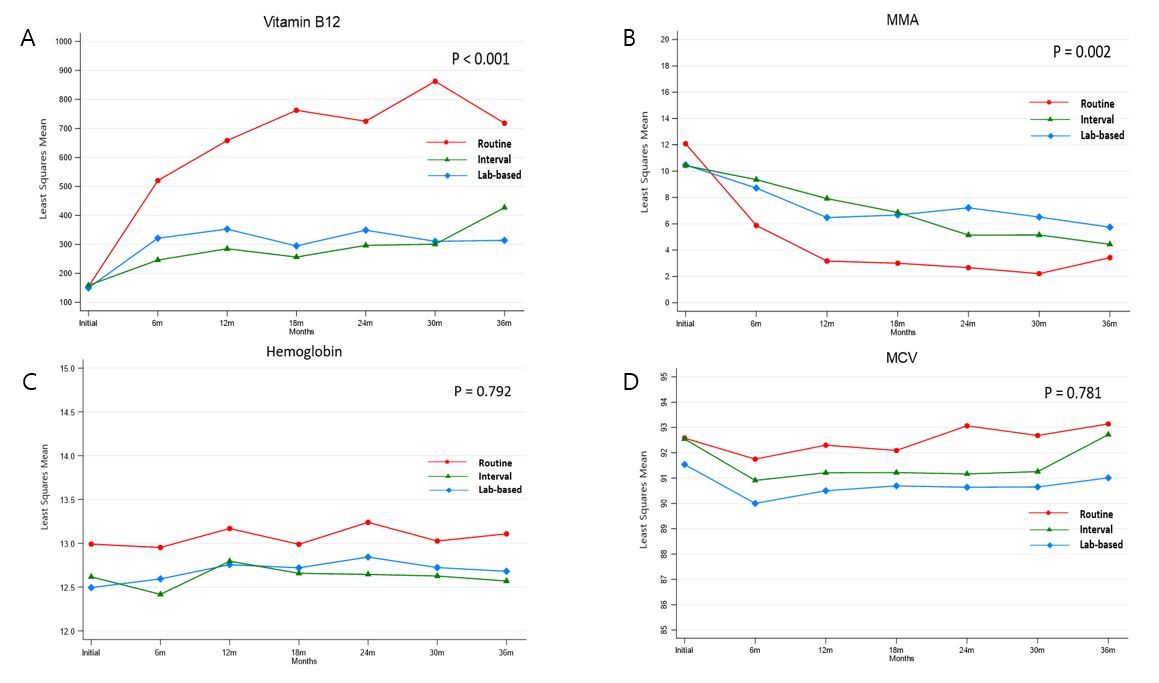

Supplement: Supplementary file 1 [file cancers-15-04938-s001.zip › Supple fig 1.JPG]
